# Assessing potential European areas of Pierce’s disease mediated by insect vectors by using spatial ensemble model

**DOI:** 10.3389/fpls.2023.1209694

**Published:** 2023-06-16

**Authors:** Sunhee Yoon, Wang-Hee Lee

**Affiliations:** ^1^ Department of Smart Agriculture Systems, Chungnam National University, Daejeon, Republic of Korea; ^2^ Department of Biosystems Machinery Engineering, Chungnam National University, Daejeon, Republic of Korea

**Keywords:** CLIMEX, ensemble modeling, insect vectors, MaxEnt, *Xylella fastidiosa*

## Abstract

Pierce’s disease (PD) is a serious threat to grape production in Europe. This disease is caused by *Xylella fastidiosa* and is mediated by insect vectors, suggesting its high potential for spread and necessity for early monitoring. In this study, hence, potential distribution of Pierce’s disease varied with climate change and was spatially evaluated in Europe using ensemble species distribution modeling. Two models of *X. fastidiosa* and three major insect vectors (*Philaenus spumarius, Neophilaenus campestris*, and *Cicadella viridis)* were developed using CLIMEX and MaxEnt. The consensus areas of the disease and insect vectors, along with host distribution, were evaluated using ensemble mapping to identify high-risk areas for the disease. Our predictions showed that the Mediterranean region would be the most vulnerable to Pierce’s disease, and the high-risk area would increase three-fold due to climate change under the influence of *N. campestris* distribution. This study demonstrated a methodology for species distribution modeling specific to diseases and vectors while providing results that could be used for monitoring Pierce’s disease by simultaneously considering the disease agent, vectors, and host distribution.

## 1 Introduction

Pierce’s disease, caused by *Xylella fastidiosa*, damages various economically important agricultural crops, including grapes, almonds, citrus fruits, coffee, and peaches (Almeida et al., 2005; European Food Safety Authority, 2020). *X. fastidiosa* was first reported in the USA (Pierce, 1892) and has spread to Europe and Asia (Leu and Su, 1993; Montero-Astua et al., 2008; Amanifar et al., 2014; Denancé et al., 2017), causing Pierce’s disease with symptoms of leaf chlorosis, wilting, and diebacks in infected plants (Almeida et al., 2005). *X. fastidios*a has been of particular significance in Europe since its initial discovery in olive trees in southern Italy (Saponari et al., 2013). This disease has severely damaged agricultural crops in European countries, including France, Spain, Portugal, and Germany (EFSA Panel on Plant Health, 2015; Olmo et al., 2017). *X. fastidiosa* is currently listed on the EPPO A2 list of pests recommended for regulation as a quarantine pest, demonstrating the need for monitoring to suppress damage and disease severity (EPPO, 2019).

*X. fastidiosa* is transmitted to other hosts through insect vectors that feed on the xylem tissue of plants (Almeida, 2016) and establish persistent and non-circulative infections within the foregut of insects (Purcell and Finlay, 1979; Almeida et al., 2005). These vectors of Pierce’s disease are found in many parts of the world and have spread the disease. In Europe, *Philaenus spumarius* is a major insect vector widely found in various habitats, including agricultural fields, grasslands, and woodland edges (Cornara et al., 2017). This pest causes significant damage to olive trees in Italy (Cornara et al., 2017). Species from the *Aphrophoridae* family, including *Neophilaenus campestris* and *Philaenus italosignus*, and species from the *Cicadellidae* family, including *Cicadella viridis*, are known vectors of *X. fastidiosa* in Europe (Trkulja et al., 2022). These vectors acquire the bacterium when they feed on infected plants and can spread Pierce’s disease by feeding on host plants. Moreover, because the flight ability of these vectors increases the risk of the spread of *X. fastidiosa* it is important to control insect vectors to prevent the damage caused by this disease, requiring a method that effectively confines potential areas exposed to insect vector distribution (Lago et al., 2021).

Species Distribution Model (SDM) evaluates the potential distribution and occurrence probability of a species as a function of the estimated relationships among species biology, occurrence areas, and environmental characteristics (Elith and Leathwick, 2009) and has been further applied for the spatial prediction of disease and surveillance of invasive species (Peterson and Vieglais, 2001; Peterson et al., 2003). Owing to its advantages in screening areas exposed to the target species in advance, it has been applied to develop the fundamentals necessary for monitoring and controlling diseases and pests (Bosso et al., 2017; Jung et al., 2019; Byeon et al., 2021; Lee et al., 2022; Song et al., 2022; Yoon et al., 2023). SDM algorithms can generally be classified into mechanistic and correlative models (Kearney et al., 2010; Li and Wang, 2013). Each algorithm differs in the required data, variable format, operational method, and process of obtaining predicted results, meaning that suitable algorithms vary according to the available information, target species, and research purpose. Recently, an ensemble model that uses two or more models has been used to complement the uncertainty of individual models and improve their reliability (Araújo and New, 2007; Kumar et al., 2015; Narouei-Khandan et al., 2020). Hence, its application to evaluate the potential distribution of a species is increasing, which has led to the development of ensemble models for studies with worldwide concerns (Araújo and New, 2007; Mainali et al., 2015). The use of multiple environmental variables, not just climatic factors, improves predictive performance (Matyukhina et al., 2014; Bradie and Leung, 2017; Lee et al., 2021).

Few studies have investigated the characteristics of Pierce’s disease in terms of symptoms, ecology, and vectors (Davis et al., 1978; Anas et al., 2008; Chatterjee et al., 2008; Raffini et al., 2020). However, SDM studies assessing the risk of the disease are relatively limited with two notable studies by using the MaxEnt model (Bosso et al., 2016a), and the CLIMEX model (Hoddle, 2004). These studies showed the notable application of SDM to the disease, but both are simple models only utilizing the disease records and climatic data, suggesting a need for considering additional environmental conditions with a recently advanced modeling technique. In this study, we evaluated the potential risk areas for Pierce’s disease in Europe using a novel ensemble SDM, integrating different algorithms used for the disease and vectors with host distribution due to climate change. CLIMEX, a mechanistic model, was used to predict climatically suitable regions for *X. fastidiosa*, while MaxEnt, a correlative presence-only model, was used to evaluate potential areas of occurrence for major insect vectors (Raffini et al., 2020). The ensemble model was then spatially constructed by projecting the consensus areas of the disease and insect vectors in addition to the host distribution so that the final outcome could identify high-risk areas vulnerable to Pierce’s disease.

## 2 Materials and methods

### 2.1 Acquisition and processing of occurrence data

The occurrence coordinates of *X. fastidiosa* were obtained from the disease distribution by using Global Biodiversity Information Facility (GBIF, 2022a), Center for Agriculture and Bioscience International (CABI, www.cabi.org), and previous studies (Bosso et al., 2016b; Castillo et al., 2019; Safady et al., 2019). A total of 49 distribution coordinates in Europe were confirmed after cross-checking the above multiple sources, which should minimize occurrence uncertainty.

**Figure 1:**
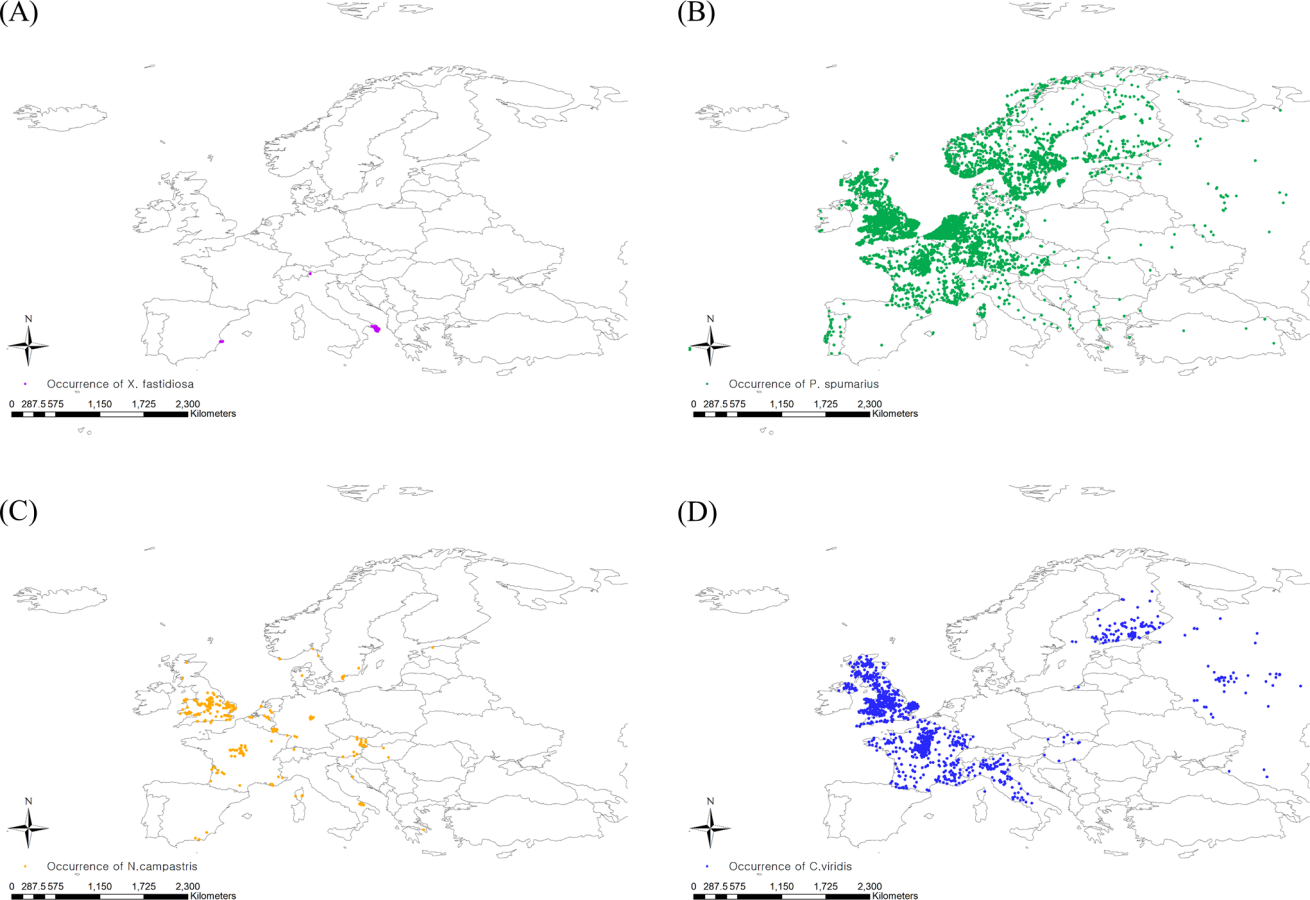
Occurrence coordinates of **(A)** Xylella fastidiosa, **(B)** Philaenus spumarius, **(C)** Neophilaenus campestris and **(D)** Cicadella viridis.

For insect vectors of Pierce’s disease, *P. spumarius*, *N. campestris*, and *C. viridis* were selected because they are the widely distributed predominant vectors of *X. fastidiosa* in Europe (Janse and Obradovic, 2010; Elbeaino et al., 2014; Morente et al., 2018). To obtain specific occurrence coordinates, we used GBIF (GBIF, 2022b; GBIF, 2022c; GBIF, 2022d) and CABI, and the occurrence data were carefully determined by cross-checking the two databases. Then, spatial filtering was applied to the occurrence data to minimize the sampling bias by balancing the sampling density (Kramer-Schadt et al., 2013). The spatial filtering buffer was determined based on flying ability, setting 5 and 3 km for *P. spumarius* and *N. campestris*, respectively, whereas *C. viridis* was spatially filtered with a default buffer radius of 10 km (Lago et al., 2021). The spatial rarefying function in the SDM toolbox developed for ArcGIS (version 10.8.1, ESRI, USA) was employed for spatial filtering (Brown, 2014). Finally, 4772, 234, and 993 European occurrence points for *P. spumarius*, *N. campestris*, and *C. viridis*, respectively, were confirmed on the map (**Figure 1**).

### 2.2 Acquisition and processing of meteorological data

Meteorological data from 1990–2018, including maximum temperature, minimum temperature, precipitation, and elevation, were obtained with a 2.5-minute resolution from WorldClim (https://www.worldclim.org) (Fick and Hijmans, 2017). The meteorological data were then converted into 19 bioclimatic variables to be used as MaxEnt model variables in ASCII format using R software (R Core Team, 2021) (Hijmans et al., 2017). The obtained meteorological data were extracted for the cells assigned to Europe and converted into two separate files, recording locations (loc file) and meteorology (met file), which was the required format for the CLIMEX model (Kriticos et al., 2015).

For future prediction, a 2.5-minute resolution of the Shared Socioeconomic Pathways (SSP) 585 climate change scenario for 2041–2060, generated by the MIROC6 model, was obtained (Fick and Hijmans, 2017). For the MaxEnt model, the scenario was obtained in the form of a bioclimatic variable identically defined to the current bioclimatic variables. In contrast, the monthly average minimum temperature, maximum temperature, and precipitation of SSP 585 were obtained for the CLIMEX model. Then, the same data processing was applied to obtain a format applicable to the CLIMEX model, as was done for the current meteorological data. Consequently, we obtained current and future meteorological data of the same type and time for use with different SDM tools.

### 2.3 MaxEnt modeling for insect vectors

MaxEnt is a modeling algorithm that we used for the three insects (Raffini et al., 2020). This model evaluates the possibility of occurrence by training environmental variables in the occurrence areas of a species (Phillips et al., 2004; Phillips et al., 2006; Elith et al., 2011). Because the spatial autocorrelation of bioclimatic variables can lead to model overfitting, it is necessary to select model variables that are not correlated. In this study, a variable showing a correlation coefficient > 0.8 for a biologically driven variable was removed (Kumar et al., 2014; Ancillotto et al., 2019; Yoon and Lee, 2021). The selected MaxEnt model variables for insect vectors were 10, 12, and 10 bioclimatic variables for *P. spumarius*, *N. campestris*, and *C. viridis*, respectively (**Table 1**).

**Table 1:** CLIMEX parameter values for *Xylella fastidiosa*.

Parameters	Code	*X. fatidiosa* ^*^
Temperature		
Limiting low temperature (°C)	DV0	5
Lower optimal temperature (°C)	DV1	12
Upper optimal temperature (°C)	DV2	34
Limiting high temperature (°C)	DV3	37
Moisture		
Limiting low soil moisture	SM0	0.1
Lower optimal soil moisture	SM1	0.5
Upper optimal soil moisture	SM2	1.75
Limiting high soil moisture	SM3	2
Cold stress (CS)		
CS temperature threshold (°C)	TTCS	-1
CS temperature rate	THCS	-0.001
CS degree-day threshold (°C)	DTCS	20
CS degree-day rate	DHCS	-0.00025
CS average temperature threshold (°C)	TTCSA	4
CS average temperature rate	THCSA	0
Heat stress (HS)		
HS temperature threshold (°C)	TTHS	34
HS temperature rate	THHS	0.001
HS degree-day threshold (°C)	DTHS	0
HS degree-day rate	DHHS	0
Dry stress (DS)		
DS threshold	SMDS	0.1
DS rate	HDS	-0.005
Wet stress (WS)		
WS threshold	SMWS	2
WS rate	HWS	0.002

^*^Parameters are from Hoddle, 2004 for X. fatidiosa.

Because it is required to determine the model features and regularization multiplier (RM), we used ENMeval in the R package, which compares all possible combinations of model settings to find the RM and the best model features of linear (L), quadratic (Q), product (P), threshold (T), and hinge (H) based on the Akaike information criterion (AIC) (Muscarella et al., 2014). The optimal model features for *P. spumarius* and *C. viridis* were RM of 0.5, and LQHPT features, while RM of 0.5 and LQ features were optimal for *N. campestris* evaluation. The model was then operated with 10,000 random backgrounds using 10-fold cross-validation, and the output was recorded in logistic format and projected onto a map using ArcGIS.

Two widely used metrics were employed to evaluate the reliability of the developed model: the area under the receiver operator curve (AUC) and true skill statistics (TSS) (Fielding and Bell, 1997; Allouche et al., 2006; Merow et al., 2013). In general, AUC < 0.7 is considered a poor performance, 0.7 ≤ AUC < 0.8 is moderate, and AUC ≥ 0.8 is good to excellent performance (Merckx et al., 2011; Peterson et al., 2011). True skill statistics, a more practical metric than the AUC, were calculated using a threshold value that maximized the sum of sensitivity and specificity (Liu et al., 2005). In general, it was considered that a value of TSS < 0.2 indicated a poor performance, 0.2 ≤ TSS < 0.4 was an acceptable performance, 0.4 ≤ TSS <0.6 was a moderate performance, and TSS ≥ 0.6 suggested a good performance (Landis and Koch, 1977; Tobeña et al., 2016).

### 2.4 CLIMEX modeling for Pierce’s disease

CLIMEX (version 4.0; Hearne Software, Melbourne, Australia) predicts the potential distribution of a species by evaluating the biologically suitable areas in a local climate (Kriticos et al., 2015). CLIMEX uses parameters representing the biological responses of a species to climate to evaluate the possibility of pest invasion based on climatic suitability (Byeon et al., 2018). The outcome is Ecoclimatic Index (EI), a quantitative representation of the climatic suitability of a species in a specific area. The EI value, which comprehensively estimates species growth and inhibition under given climatic conditions, was scaled from 0 to 100 (Kriticos et al., 2015). A species cannot be theoretically established at zero EI, whereas an EI >30 suggests an optimal climate for species inhabitation (Kriticos et al., 2015). In this study, we employed a previously developed CLIMEX model for Pierce’s disease (Hoddle, 2004) (**Table 2**).

**Table 2:** Model performance and variables that contributed to the MaxEnt model.

Philaenus spumarius			Neophilaenus campestris			Cicadella viridis		
AUC	0.79		AUC	0.94		AUC	0.90	
TSS	0.84		TSS	0.89		TSS	0.91	
Variable	Percent contribution	Permutation importance	Variable	Percent contribution	Permutation importance	Variable	Percent contribution	Permutation importance
Temperature Seasonality (Bio4)	65.6	58.1	Min Temperature of Coldest Month (Bio6)	36.3	2.8	Isothermality (Bio3)	39.3	26.5
Precipitation of Warmest Quarter (Bio18)	9	0.7	Isothermality (Bio3)	17.4	2.7	Temperature Annual Range (Bio7)	16.8	13.3
Annual Mean Temperature (Bio1)	7.4	4.8	Temperature Seasonality (Bio4)	17.2	49.9	Precipitation of Warmest Quarter (Bio18)	10.7	9.1
Max Temperature of Warmest Month (Bio5)	7.4	16.2	Precipitation Seasonality (Bio15)	9.5	0.1	Annal Mean Temperature (Bio1)	10.7	16.8
Elevation	5	7.4	Elevation	6.5	1.8	Mean Temperature of Driest Quarter (Bio9)	6.4	4.3
Mean Diurnal Range (Bio2)	2	2	Mean Temperature of Driest Quarter (Bio9)	3.3	4	Precipitation Seasonality (Bio15)	3.6	5.5
Isothermality (Bio3)	1.3	4.3	Precipitation of Wettest Quarter (Bio16)	2.7	0.3	Mean Temperature of Warmest Quarter (Bio10)	3.5	7.5
Mean Temperature of Driest Quarter (Bio9)	1	4.2	Mean Temperature of Driest Quarter (Bio8)	2.4	1.7	Mean Temperature of Driest Quarter (Bio8)	3.1	1.8
Precipitation Seasonality (Bio15)	0.6	0.9	Precipitation of Driest Month (Bio14)	1.6	0.1	Elevation	3.1	1.9
Mean Temperature of Driest Quarter (Bio8)	0.5	0.6	Temperature (Bio1)	1.3	11.7	Mean Diurnal Range (Bio2)	1.4	5.1
Precipitation of Wettest Month (Bio13)	0.2	0.9	Mean Temperature of Warmest Quarter (Bio10)	1.1	24.7	Precipitation of Wettest Month (Bio13)	1.4	8.3
			Precipitation of Wettest Month (Bio13)	0.4	0			
			Mean Diurnal Range (Bio2)	0.3	0.2			

Because of the characteristics of the CLIMEX model, which determines the parameter sets showing the best fit to the actual distribution data, there is no standard method for evaluating the performance of the CLIMEX model. Therefore, we estimated its accuracy by counting the actual occurrence records included in the simulation, as in previous CLIMEX studies (McConnachie et al., 2011; Saavedra et al., 2015).

### 2.5 Distribution of host of *X. fastidiosa*

**Figure 2:**
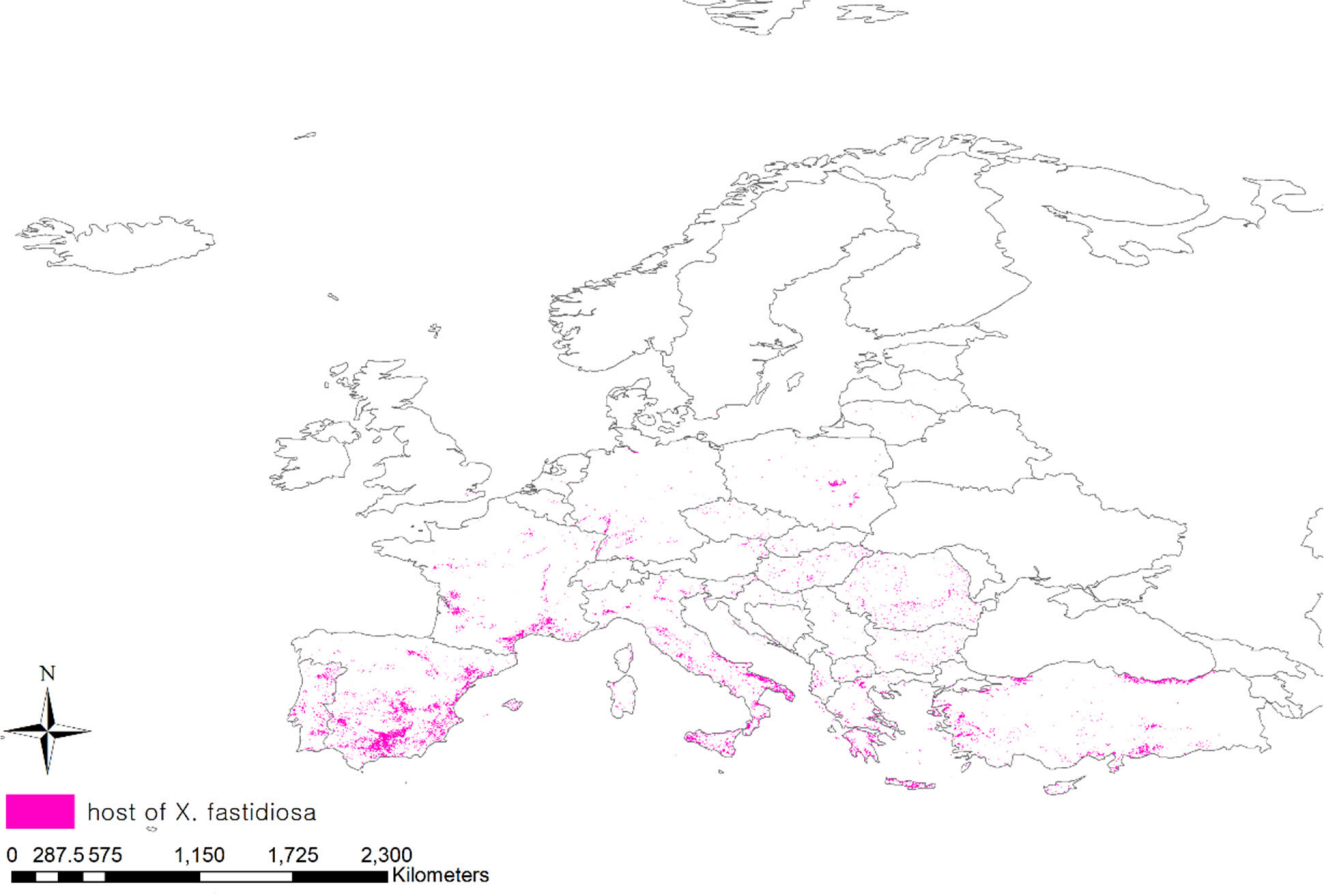
Distribution area of host of *Xylella fastidiosa*.

Pierce’s disease affects more than 300 plant species, including grapes, citrus fruits, coffee, olives, almonds, blueberries, and other herbaceous plants (Stancanelli et al., 2015). To consider the distribution of host plants, we obtained a geodatabase of land cover maps in Europe using the Copernicus Land Monitoring Service (CLC, https://land.copernicus.eu/) (Büttner, 2014). Among the 44 classified areas in the land cover, areas planted with vineyards, fruit trees, berry plantations, and olive groves that were particularly damaged by Pierce’s disease, were extracted and projected onto the map (**Figure 2**).

### 2.6 Ensemble mapping of the potential distributions of Pierce’s disease and insect vector

The current and future potential distributions of insect vectors and the climatic suitability for Pierce’s disease predicted using MaxEnt and CLIMEX, respectively, were overlaid using ArcGIS (Byeon et al., 2021; Lee et al., 2021). The prediction of insect vectors was converted into binary maps by establishing a common threshold value (the 10^th^ percentile training presence logistic threshold in MaxEnt) to classify presence or absence. The binary map of each insect vector was then superimposed on a scale of cells (the minimum projection unit under a given resolution) to find the consensus areas for all three insect vectors in Europe. The CLIMEX result of Pierce’s disease was also converted into a binary map divided into suitable regions with EI ≥1 and unsuitable regions with EI<1. The two binary maps were overlapped to define consensus areas showing the potential distributions of both the disease and insect vectors. The regions that were potentially the most vulnerable to Pierce’s disease were identified by overlapping the host distribution map with the disease vector map.

## 3 Results

### 3.1 Evaluating potential distribution of Pierce’s disease using CLIMEX

**Figure 3:**
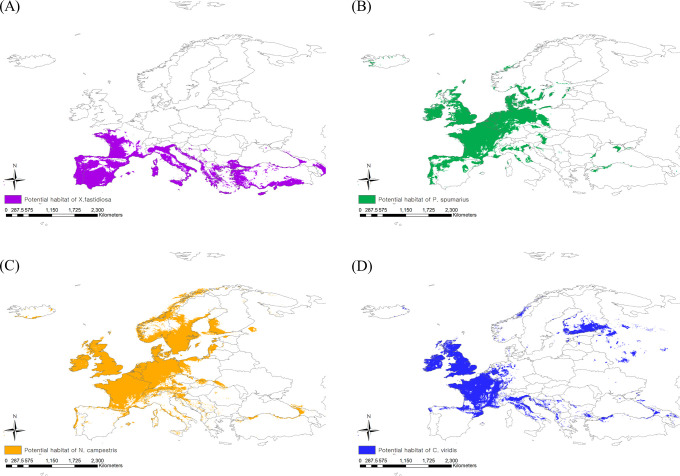
Potential risk area under the current climate of **(A)** *Xylella fastidiosa*, **(B)** *Philaenus spumarius*, **(C)** *Neophilaenus campestris* and **(D)** *Cicadella viridis*.

The existing CLIMEX model includes all occurrence coordinates of *X. fastidiosa* in Europe within the prediction region, suggesting that the model is reliable (Hoddle, 2004). When applying a threshold level of EI > 1, *X. fastidiosa* was predicted to be distributed in the southern regions of Europe under the current climate, with an estimated area of 1,948,597 km^2^ (**Figure 3A**). However, it is predicted to increase to 3,137,960 km^2^, reaching Hungary, England, Belgium, and Germany, in future based on climate change.

### 3.2 Evaluating the potential distribution of insect vectors of Pierce’s disease using MaxEnt

The developed MaxEnt models for insect vectors showed AUC values of 79, 0.94, and 0.90, and TSS values of 0.84, 0.89, and 0.91 for *P. spumarius*, *N. campestris*, and *C. viridis*, respectively, suggesting the model performance was sufficient.

**Figure 4:**
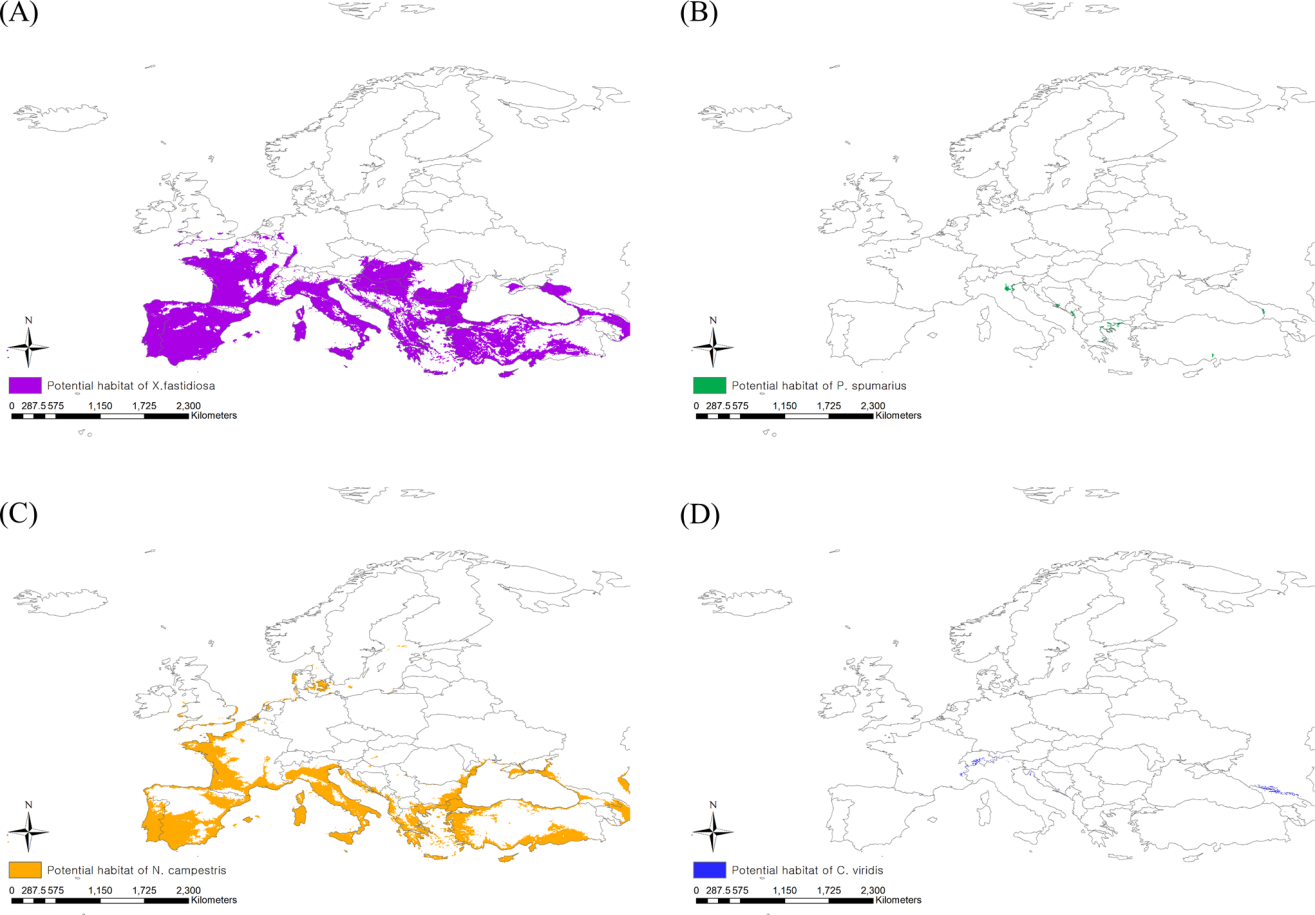
Potential risk area under the future climate (2041–2060) of **(A)** *Xylella fastidiosa*, **(B)** *Philaenus spumarius*, **(C)** *Neophilaenus campestris* and **(D)** *Cicadella viridis*.

When applying the 10^th^ percentile training presence logistic threshold under the current climate, the potential distribution areas of *P. spumarius* were estimated to be 2,333,408 km^2^ including France, Germany, Belgium, the Netherlands, the United Kingdom, some neighboring countries, and the northern area of Turkey (**Figure 3B**). The largest potential distribution areas were observed for *N. campestris* at 3,460,361 km^2^, reaching northern Europe (Denmark, Sweden, Norway, and Finland) (**Figure 3C**). The potential distribution area of *C. viridis* was estimated to be 1,854,718 km^2^, covering France, the United Kingdom, Finland, and eastern Russia (**Figure 3D**). With climate change, the potential distribution areas of the three insect vectors tended to shift southward, but their sizes decreased drastically (**Figures 4A–D**). The potential distribution area of *P. spumarius* was observed only in small spots in Italy, Croatia, Albania, Greece, and Georgia, totaling 26,487 km^2^, which is a 98% decrease from the area under the current climate. *N. campestris* is expected to move southward to Spain, Italy, Greece, and Turkey. However, the area decreased to 1,895,461 km^2^, equivalent to 55% of the current area. The potential distribution area of *C. viridis* shrank to 14,358 km^2^, which is approximately 99% less than the potential area under the current climate and showed small spots in Italy, Greece, and Georgia.

Bioclimatic variables related to climatic variation contributed to the model performance, suggesting that these pests are sensitive to climatic conditions (**Table 1**). Temperature seasonality showed the highest contribution (65.6%) to the model for *P. spumarius*, followed by precipitation in the warmest quarter (9%) and annual mean temperature (7.4%). For *N. campestris*, the minimum temperature of the coldest month showed the highest contribution (36.3%), followed by isothermality (17.4%) and temperature seasonality (17.2%). Isothermality contributed the most (39.3%) to the model of *C. viridis*, followed by annual temperature range (16.8%) and precipitation of the warmest quarter (10.7%).

### 3.3 Evaluating the potential risk areas of Pierce’s disease damage by using ensemble mapping

**Figure 5:**
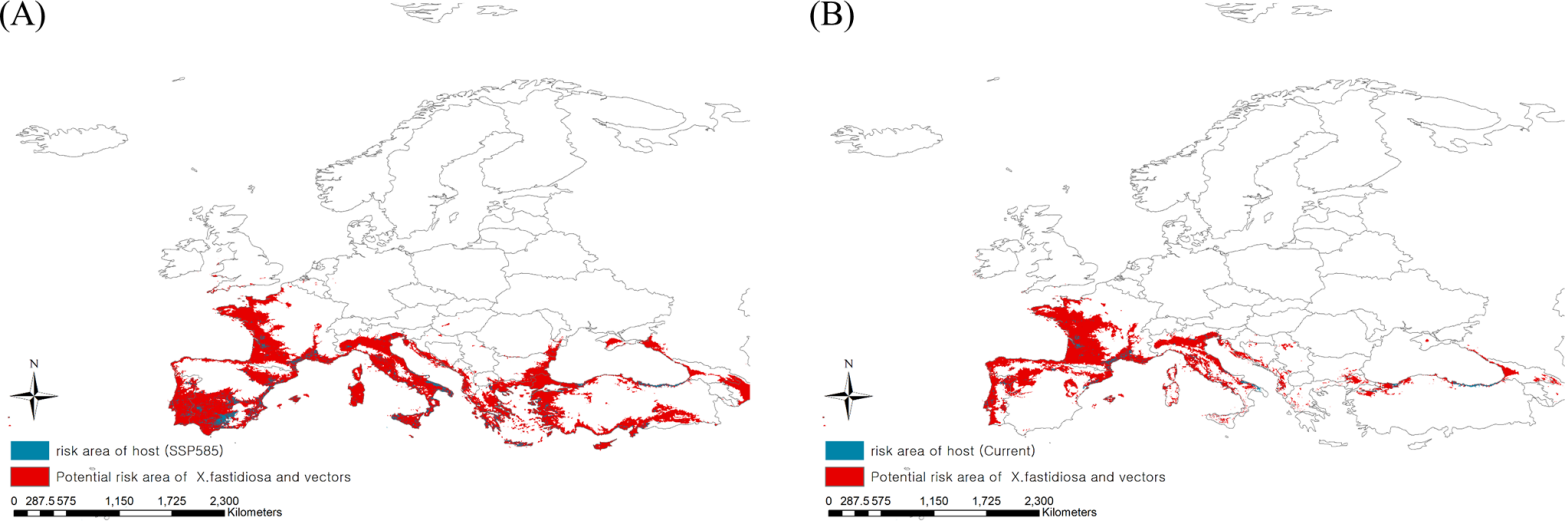
Ensemble mapping to illustrate high risk areas of Pierce’s disease mediated by insect vectors under the **(A)** current and **(B)** future climate (2041–2060).

High-risk areas where Pierce’s disease could be mediated by insect vectors were derived by extracting the consensus areas for Pierce’s disease and insect vectors under current and future climates (**Figure 5**). The present high-risk areas, which is estimated to be 849,062 km^2^, were in southern Europe, such as Spain and Italy, as well as in eastern France. Based on climate change, risky areas could expand to 1,731,618 km^2^, covering most regions of southern Europe, such as Portugal, Spain, Italy, Greece, and Turkey. This is because the consensus areas increased as the potential area for Pierce’s disease moved southward, although the potential distribution area of the vectors decreased. 

Three maps projecting the disease, insect vectors, and hosts were constructed to identify the most threatened host area (**Figure 5**). Because of the southward expansion of potential areas of Pierce’s disease, the host distribution region was estimated to be affected more than three-fold due to climate change: 121,637 km^2^ in the future from 36,082 km^2^ under the current climate of 149,286 km^2^ of host distribution.

## 4 Discussion

This study used dual-species distribution modeling of mechanistic and correlative algorithms for an ensemble spatial analysis of the potential distribution of Pierce’s disease. Ensemble models have recently been used to build reliable models by combining the characteristics of different algorithms in cases with many uncertainties, such as in species distribution modeling (Stohlgren et al., 2010). The decision on which type of algorithm to apply to each species should be based on the characteristics of the target species, the amount of available data, and the purpose of the prediction (Yoon et al., 2023). Previously, it was shown that SDM was applicable for evaluating risk areas exposed to *X. fastidiosa* distribution according to climate change (Bosso et al., 2016a). However, the model was developed using the limited coordinates and outdated climatic variables. Because the correlative SDM highly depends on occurrence coordinates, the limited number of *X. fastidiosa* occurrence records might increase uncertainty of a modeling result. In addition, the correlative SDM is trained by values assigned to variables at occurrence coordinates, meaning the variables that can reflect the current conditions can increase the model reliability. Thus, it is necessary to compensate these drawbacks by employing additional conditions and updated variable information. This attempted to decrease model uncertainty with the ensemble modeling of the disease and insect vectors as the disease spread is constrained by habitats of them, and to increase model reliability with the updated climatic variables recorded by 2018. This study focused on identifying areas exposed to the risk of Pierce’s disease mediated by insect vectors. Thus, we aimed to construct a model that incorporates the habitats of actual vectors with biologically possible regions of disease occurrence, along with the availability of occurrence data. The climate is an important factor affecting the outbreak of Pierce’s disease, as the viability of Pierce’s disease varies with weather conditions, in addition to the breed or age of the infected host (Feil and Purcell, 2001). Tropical, subtropical, and Mediterranean climates favor the survival and development of Pierce’s disease, implying that the probability of the disease occurring is high under favorable climatic conditions through vector mediation (Purcell, 1997). For this reason, the CLIMEX model might be more suitable because it constructs a physiological niche by analyzing the suitability within a given climate based on the biological characteristics of a species, compared with a machine learning-based model that finds a realized niche that is environmentally similar to the occurrence area (Kriticos et al., 2015). In contrast, the MaxEnt model was used for insect vectors because a relatively sufficient number of occurrence coordinates was available, which is the most important requirement in developing a reliable machine learning-based model (Phillips et al., 2004; Elith et al., 2011). Moreover, vectors are affected by other factors such as host and topology; thus, a model incorporating different variables other than climate would be suitable. We believe that ensemble models, which combine different models depending on data availability and the main targets of prediction, can increase the reliability of predictions, as opposed to simply applying the same algorithm to two or more species or applying different algorithms to one species.

The climate is a dominant factor affecting the distribution of insect species (Björkman and Niemelä, 2015). In our analysis, some areas shared the potential distribution of all three insect vectors, indicating a common climatic factor that confined their habitats. Isothermality (Bio3) contributed significantly to the MaxEnt model of the three insect vectors, which quantified the ratio of the annual maximum and minimum temperature differences to the monthly average daily temperature difference (O’donnell and Ignizio, 2012). All insect vectors were highly likely to occur at approximately 30% or higher, suggesting that small seasonal differences favored their occurrence of the insect vectors. The three species used in this study are mainly distributed in the southern and western regions of Europe, where the isothermality is high enough to aid the spread of *X. fastidiosa*. The annual temperature range in Europe is between approximately 15 and 25°C, and this range is included within the maximum and minimum temperatures (5–28°C) observed in the actual outbreak area of Pierce’s disease. This indicates that favorable conditions for the disease and vectors are consistent (Yoon and Lee, 2023). Interestingly, *N. campestris* was the only one of three vectors that could potentially mediate Pierce’s disease under climate change, while the habitats of two other species in Europe were predicted to decrease significantly. This may be due to the differences in the dominant factors that contributed the most to the model. In the model of *N. campestris*, the minimum temperature of the coldest month (Bio6) showed the highest contribution. It was found that the probability of occurrence was high in areas where Bio6 was 0 or more, and the occurrence area appeared to move to the Mediterranean coast where Bio6 was above 0 due to climate change. *N. campestris* overwinters as eggs and develops into larvae in spring; therefore, warm temperatures due to global warming may lead to the early hatching and development of the pest (Elbeaino et al., 2014). This is consistent with a previous SDM study which showed that the lowest temperature in the coldest month was a main explanatory variable for *X. fastidiosa* distribution (Raffini et al., 2020). This suggests that winter climate not only serves as an important model variable for predicting the spread of diseases mediated by insect vectors but also indicates its significance as a factor in forecasting disease outbreaks necessary for implementing control measures. In contrast, temperature seasonality (Bio4), a major variable of *P. saltuarius*, decreased with climate change, leading to an unsuitable environment for pest occurrence, whereas *C. viridis* was expected to decrease because of a decrease in isothermality (Bio3) due to climate change.

Among the hosts affected by Pierce’s disease, vineyards and olive groves play a significant role in Europe, a major wine-producing country (Bisson et al., 2002). The optimal temperature for growing grapes is 25–32°C, and they are largely cultivated near the Mediterranean coast (Hochberg et al., 2015; Gutiérrez-Gamboa et al., 2021). Temperatures below 12°C and above 34°C limited the growth and survival of *X. fastidiosa*, which is similar to the optimal temperature for growing grapes (Feil and Purcell, 2001). Growth Index (GI) is an indicator of population growth potential during the favorable season in the CLIMEX model, showing high values in the spring and fall seasons by avoiding hot and dry environments that can delay symptoms (Feil and Purcell, 2001). Although there are differences depending on the vector biology, adult emergence occurs between April and June or between July and October (Bodino et al., 2019). This is similar to the peak GI period, worsening the spread of Pierce’s disease; consequently, it is necessary to pay particular attention during this period. Pierce’s disease eventually occurs in an environment where both the host and vector are available. Unfortunately, the favorable climate and time for growing hosts are consistent with the requirements for disease and vector development. From this point of view, countries along the Mediterranean coast and western France are at high risk of Pierce’s disease occurrence regardless of climate change, whereas southern Spain is projected to become the most at-risk region for the disease due to climate change. Consequently, intensive pest and disease control before disease outbreaks outbreak is necessary for these countries (Janse and Obradovic, 2010; Raffini et al., 2020; Morelli et al., 2021).

## 5 Conclusion

This study applied different algorithms for disease and insect vectors and predicted the potential occurrence areas of Pierce’s disease that vary with climate change using an ensemble modeling approach by spatially relating the model results with the host distribution. The main occurrence areas in Europe were predicted to be near the Mediterranean coast. However, it could expand southward, mediated by insect vectors due to climate change, causing severe damage due to consistency between areas of disease occurrence and host cultivation. Therefore, it is possible to effectively identify high-risk areas for the potential occurrence of the disease in advance and to implement intensive monitoring and control to suppress the spread of the disease and minimize the potential damage that may increase due to climate change. From the perspective of SDM, this study is significant because it predicts Pierce’s disease and it methodologically proposes an ensemble model by integrating individual models of disease and vectors with the host as a model variable. Although this model does not consider changes in host distribution owing to climate change, integrating a model that considers host changes can further enhance its reliability.

## Data availability statement

The raw data supporting the conclusions of this article will be made available by the authors, without undue reservation.

## Author contributions

SY: conceptualization, methodology, software, validation, formal analysis, investigation, data curation, writing –original draft, and visualization. W-HL: conceptualization, methodology, resources, writing - review & editing, supervision, and funding acquisition. All authors contributed to the article and approved the submitted version.
